# Case of Acute Metritis Treated Successfully with Urate of Ammonia

**Published:** 1852-08

**Authors:** C. F. Percival


### *Case of Acute Metritis treated successfully with Urate of Ammonia.* By Dr. C. F. Percival.

This article appears to be advancing in public attention, since a communication on its value in this Journal, in regard to Synovial and Serous Inflammations, as the following communication will exhibit.

It appears, also, that it has been tried advantageously in chronic cutaneous diseases and in tubercle of the lung.* This reminds us that it was once the practice in Switzerland to treat consumptives by a residence over the stalls of animals.

*Benton, Lowndes Co., Alabama,* May 30th, 1852.

Professor Horner:

*Dear Sir,*—On the 25th of April I was called to Dinah Brown, a negress belonging to J. T. Brown, nine miles from my house. On the 15th of March she was delivered of a healthy child. She did well during the month, and on the 19th of April went into the field to labor. She there was caught in the violent storm which occurred on that eve, and walked home fatigued and very wet. About midnight she was taken with violent pains in the uterine region, and about 4 had a violent and excessive hemorrhage. She, however, rallied under the treatment of the plantation midwife, and got rather better, until the 23d, when I was sent for, she being worse. I found her perfectly prostrated, pulse 135, skin hot and dry, at times cold, clammy perspiration breaking out; countenance anxious. Auscultation revealed a creaking sound over uterus, which, enlarged almost to four times its size, was scarcely to be touched from the excessive pain the most careful examination produced; her bowels costive. I placed her under the use of mercurials and opium in two grain doses every three hours, opening the lower bowel by injection of turpentine suspended in white of egg and mucilage, and applied a blister over the whole abdomen. I saw her every few hours, and paid her all the attention a case of this kind requires, occasionally altering my prescriptions. The next morning, the blister having been previously dressed, the tenderness had not decreased much, but she being more comfortable and the secretions having assumed a healthier action, I applied a poultice consisting of our red earth well saturated with urine. I at that time looked on the case as hopeless, although the woman *was* a little better. On Sunday she was improving, and I continued the use of the urinated earth. The next day I left for Virginia, where I had the pleasure of meeting you, but not knowing the result of my case, would not tell it.

On my arrival home I found her well, though still weak. She

* Brit. and For. Med. Chir. Review, July, 1852; from Bucknor's Report, No. 19.

is up and spinning; she had some of the worst symptoms after I left, which were promptly relieved by injections. So much for the Urate of Ammonia. I have often been puzzled at the success I have met by using fresh cow dung poultices in cases of diseases of the knee joint, and have from motives of *fear* kept from relating cases of white swelling which I know were relieved by its use. I first learned it in the County of Armagh in Ireland; that it is of great benefit I know. And that the woman Dinah owes her life to the fact of my having read your paper on Urate of Ammonia in that knee case, is as true. And it does sometimes gladden the heart of a physician to know his efforts have been instrumental in doing good; and as the laurel belongs to you and not to me, I send the case to you, and pray for your prosperity on earth and future happiness in a better and brighter world.

Yours, affectionately,
C. F. PERCIVAL.

